# ‘They Just Said It Was My Mood. I Was Trying to Get Attention’: Exploring Barriers to Psychological Support for People Impacted by Contaminated Blood in England

**DOI:** 10.1111/hex.70317

**Published:** 2025-06-10

**Authors:** Eva Cyhlarova, Jessica Carlisle, Emily Warren, Martin Knapp, Ellen Nolte

**Affiliations:** ^1^ Care Policy and Evaluation Centre, London School of Economics and Political Science London UK; ^2^ Department of Health Services Research and Policy, London School of Hygiene & Tropical Medicine, Division of Psychiatry University College London London UK

**Keywords:** barriers, contaminated blood, hepatitis C, human immunodeficiency virus (HIV), mental health, psychological support

## Abstract

**Objectives:**

Between the 1970s and the early 1990s, over 30,000 individuals in the United Kingdom were infected with human immunodeficiency virus (HIV) and/or hepatitis C virus following treatment with NHS‐supplied blood and blood products, with devastating consequences. This study aims to better understand the psychological support needs of these individuals and their families, and to identify barriers to accessing support in England.

**Methods:**

Forty‐one individuals infected with HIV and/or hepatitis C virus and 11 affected family members were interviewed, as well as 14 mental health practitioners and experts involved in psychological support services across the United Kingdom. Data were analysed using a thematic approach.

**Results:**

Only a few infected and affected participants had received mental health support, and only just over half knew about the availability of funding for psychological support from the England Infected Blood Support Scheme. Participants identified a number of barriers preventing them from accessing support. These included personal and social factors such as family responsibilities, stigma and secrecy. Structural barriers to access were a lack of available mental health support, limited understanding among professionals of the contaminated blood scandal, discrimination in healthcare, and difficulties finding suitable therapists and navigating referral systems. When individuals managed to access support, it was often perceived as inadequate or ineffective. Practitioners also identified a substantial need for psychological support within the infected and affected communities, and described support provided as inadequate, with little guidance and limited availability of competent practitioners. Practitioners also emphasised the need for long‐term and tailored treatment approaches to address the profound mental and physical health impacts of infected blood.

**Conclusions:**

Existing psychological support systems in England, both public and private, fail to meet the needs of infected and affected communities. Our findings show a substantial and increasing need for accessible, effective and individualised services.

**Patient or Public Contribution:**

This study was carried out from August 2022 to August 2023, during the period when the statutory Infected Blood Inquiry was conducting public hearings and soliciting witness statements from people infected or affected by the contaminated blood scandal. A key consideration of our work was therefore the potential additional burden on participants who were asked to reflect on their experiences as survivors and/or bereaved family members of infected individuals within the broader context of the Inquiry. We were aware of the emotional weight this might place on participants. To address this, we collaborated with several organisations supporting infected and affected individuals in autumn 2022: the British Red Cross, the Haemophilia Society, the Hepatitis C Trust, the Terrence Higgins Trust, and the Haemophilia & Bleeding Disorders Counselling Association. Representatives of these organisations included individuals who had been infected themselves, as well as mental health practitioners. We held extensive discussions with these organisations on the contaminated blood scandal, the experiences of those impacted, and the support available. They also provided feedback on our draft research materials (information sheet and interview topic guide), which we incorporated into our final versions, and reviewed our findings. In addition, these organisations also acted as facilitators to engage infected and affected people to participate in the study, where this was possible.

## 1 Introduction

Thousands of people in the United Kingdom have experienced devastating repercussions from receiving blood or blood products contaminated with human immunodeficiency virus (HIV) and/or hepatitis C virus during the 1970s and 1980s. Although concerns about risks of infection with hepatitis from blood products were raised as early as the mid‐1970s, and for HIV from the early 1980s, contaminated blood products continued to be used into the 1990s [1]. As a result, at least 30,000 individuals were infected with HIV and/or hepatitis C following treatment with National Health Service (NHS) blood and blood products between 1970 and 1991 [2]. The majority were infected with hepatitis C following blood transfusions, and about 1250 people with bleeding disorders were infected with HIV, including 380 children [3], often also contracting hepatitis C. Between 1970 and 2019, at least 2900 deaths have been attributed to these infections [4], and people are still dying. Furthermore, some individuals infected with hepatitis C may still be unaware of their infection today [5]. Indeed, the number of infected individuals is unknown, as is the number affected by the infections, such as partners, children or parents, many of whom have been bereaved.

Available research has mainly focused on the physical health outcomes for people infected through contaminated blood and blood products, including premature death [6]. In contrast, there is limited evidence on the psychological impacts of infection [7, 8]. Evidence on the mental health impacts of receiving contaminated blood shows higher rates of depression and anxiety and lower quality of life, yet these represent just a fraction of the complex physical, emotional and psychological challenges that infected individuals must manage [7].

In the United Kingdom, the 2007 Archer Inquiry highlighted the need for psychological support and counselling for people affected by the contaminated blood scandal [9]. In response, limited funding for counselling and psychological support was made available from 2011 [10]. However, the more recent UK‐wide Infected Blood Inquiry (‘Inquiry’) [11], launched in 2018, highlighted that most infected or affected people have not had access to dedicated psychological support, ‘despite having had to struggle with the corrosive effects of infection, the debilitating side‐effects of treatment […], and the brutal manifestations of stigma’ [12] (p. 2).

The England Infected Blood Support Scheme (EIBSS), administered by the NHS Business Services Authority (NHSBSA), provides payments for psychological support (e.g., counselling or talking therapy) to eligible registered people [13]. The payment of £900 per year is available upon application and can be renewed after review. By April 2023, just over 3500 people were registered with the EIBSS, mostly infected people, 20% bereaved partners and just under 3% dependents [14]. However, in 2021/22, fewer than 5% applied for counselling payments [15].

The reasons for the low uptake of psychological support payments are unclear. The Inquiry noted that the annual amount and complex application process might partially explain the low uptake [16]. Our study aimed to better understand the psychological support needs and the barriers that individuals and families impacted by infected blood encounter when accessing psychological support in England.

## 2 Methods

We conducted a descriptive qualitative study using in‐depth interviews with (i) individuals affected by HIV and/or hepatitis C virus and/or their family members in England, and (ii) mental health practitioners and experts involved in psychological support services across the United Kingdom. We reviewed documents on support services in Northern Ireland, Scotland and Wales to understand their approaches.

We consulted with organisations supporting impacted individuals (the British Red Cross, the Haemophilia Society, the Hepatitis C Trust, the Terrence Higgins Trust, and the Haemophilia & Bleeding Disorders Counselling Association) to shape the work. These organisations provided feedback on research materials and helped recruit participants.

### 2.1 Sampling and Recruitment

#### 2.1.1 Infected and Affected People

Between March and May 2023, we recruited participants using two routes. First, the support organisations advertised our study to their subscribers, or through word of mouth, using snowball sampling. Out of 36 infected individuals or family members who expressed interest, 24 were interviewed. We did not interview two participants as they were not from England and therefore not eligible to receive EIBSS support. Ten people who initially expressed interest decided not to take part. Second, we used the 2022 Service Satisfaction Survey of EIBSS beneficiaries, which included questions on psychological support. Interested respondents provided contact details, and NHSBSA shared their demographic data. Out of 366 interested respondents (20%), we purposively contacted 63 to capture diverse demographic backgrounds and experiences with EIBSS payments and psychological support; 28 agreed to be interviewed (Table 1). More details on recruitment can be found in Cyhlarova et al.'s study [15]

**Table 1:** Sampling of interview participants recruited through the England Infected Blood Support Scheme (EIBSS) survey.

	Interviewed
Has received discretionary payment and has had psychological treatment in the past	6
Aware of but has not received discretionary payment and has had psychological treatment in the past	4
Not aware of the discretionary payment and has had psychological treatment in the past	3
Has not had psychological treatment in the past and does not wish to access treatment^a^	4
Has not had psychological treatment in the past and does not know whether they wish to access treatment^a^	3
Has not had psychological treatment in the past and wishes to access treatment^a^	5
Did not wish to disclose survey responses to the research team	3
Total	28

^a^ Response to the question ‘Would you or a family member want to have access to any psychological treatment, support or counselling linked to your, or your partner's, infection?’ (No, Don't know and Yes).

Participants were contacted by a researcher who provided information about the study and consent forms. Written consent was confirmed verbally at the start each interview. Participants received £35 payment (vouchers or cash) and travel expenses.

#### 2.1.2 Mental Health Practitioners and Experts

We contacted mental health practitioners and experts from various organisations in England and the devolved nations through websites, networks and recommendations. Out of 19 invited, 16 expressed interest and 14 were interviewed.

### 2.2 Data Collection

Interviews followed a semi‐structured topic guide based on consultations with experts and support organisations. For infected and affected individuals, we explored the impact of their infection(s), their views and experiences of psychological and other support, and their experiences with the EIBSS. We also collected demographic information. Interviews were conducted online (*n* = 31), in‐person (*n* = 12) or by telephone (*n* = 9) according to participant preference, and recorded upon consent. One interview involved two participants (a couple). Interviews were conducted by E.C. and J.C.; they lasted an average of 60 min.

Interviews with mental health practitioners and experts explored the current need for psychological support, barriers to access and the characteristics of effective support services. The topic guide was adapted to reflect differences in services across the United Kingdom. Interviews were conducted by E.N.; they were recorded using MS Teams and lasted 52 min on average.

Interview guides are available in Supporting Information File 1, and further information in the Consolidated criteria for reporting qualitative research checklist (Supporting Information File 2).

### 2.3 Analysis

Interview recordings were professionally transcribed, anonymised and analysed thematically for each group (i) infected and affected people and (ii) mental health practitioners and experts) using NVivo 2020 [17]. The research team read and reread the transcripts and discussed them to identify relevant themes. Initial coding by E.W. was based on the research questions and objectives and was refined into a parent–child node hierarchy through inductive and axial coding, exploring relationships between in vivo codes. The team met regularly to discuss and identify patterns of shared meanings or ‘central organising concepts’ using an open and iterative approach [18, 19]. The process of developing, synthesising and contrasting themes continued until the final analysis [20] and a rich interpretation of the meaning and experience of the contaminated blood scandal [21]. The analysis used a thematic approach, incorporating elements of grounded theory, such as constant comparison and the examination of deviant cases.

### 2.4 Ethical Review

Ethical approval for this study was granted by the Observational/Interventions Research Ethics Committee at the London School of Hygiene & Tropical Medicine (LSHTM Ethics Ref: 28215).

## 3 Results

We present our analysis of interviews with 41 infected individuals, 11 family members (10 bereaved) and 14 mental health practitioners and experts. Most of the infected and affected participants were aged over 50, identified as White British, and 32 were female (Table 2).

**Table 2:** Demographic characteristics of infected and affected people participating in interviews.

	*N*		*N*
Infected	41	*Ethnicity*	
Affected	11	Black/Black British, African	1
Bereaved	10	Asian/Asian British	4
Gender		Mixed: White and Asian	1
Male	20	Mixed: White Other	1
Female	32	White: British	37
Age group		White: Irish	1
21–30	1	White: European	2
31–40	3	White: Other	3
41–50	8	British	2
51–60	17	*Region of residence (current)*	
61–70	13	East of England	7
71+	10	London	7
Recruitment route		South East	12
EIBSS Survey	28	South West	7
Other*	24	Midlands	8
		North West	4
		Yorkshire/Humber	5
		Wales	1
		Europe	1

* People volunteering for an interview after receiving information about the study (via other organisations or word of mouth), including *n* = 3 recruited from the EIBSS Survey.

Our analysis identified three overarching themes that describe barriers to accessing psychological support from the perspective of infected and affected people and mental health practitioners: personal and social barriers, structural barriers, and barriers relating to the EIBSS. Before discussing these, we briefly reflect on the need for psychological support among infected and affected individuals to set the context for the diverse barriers encountered when accessing support. Identification numbers starting with ‘1’ refer to infected people, with ‘2’ to affected people and with ‘3’ to mental health practitioners and experts.

### 3.1 Setting the Context: Impact of Infection and Need for Psychological Support

Many participants reported trauma, grief, loss, anger, anxiety, guilt, stigma and isolation. They frequently mentioned long‐term health issues and significant emotional and financial consequences of bereavement. Many described coping with ongoing severe psychological distress on their own, often trying to get on with their lives, despite struggling from the lasting effects of their infections and fears about the future. Some voiced concerns about growing older and depending on healthcare, exacerbated by their negative experiences of care in the past.

As I get older, I'm fearful for my life, I'm fearful of what I'd be in this country and the way we treat, let alone someone who is HIV positive, HIV‐positive, but how we treat the elderly. […] I have no children. I have no one. I'm fearful of that vulnerability that I know because I've experienced it and I've been there. I've been down to six stone, I've been dying, I've been emaciated. I've been—and I know what it's like to be in a social, in a healthcare system with no one.

[101]

Participants reported devastating consequences of their infections, including loss of livelihoods and homes and damage to marriages, family relationships and social networks. Most infected individuals described profound impacts on their own and their loved ones' mental health. Most had endured extremely challenging periods, including severe breakdowns, suicidal thoughts, depression, flashbacks, feelings of worthlessness and intrusive thoughts.

And I wasn't there for [my children]; I wasn't there for them. And that is the one thing that I feel so guilty about. And they're grown up now […] and I never told them about what I had [hepatitis C]. So, all they saw was this woman who was like doing whatever she wanted to do and going to bed and not able to cope with them. And I think they've had terrible problems.

[107]

Mental health practitioners and experts emphasised the diversity of psychological support needs. It was noted that some communities had been dealing with the consequences of infection for decades, while others, recently diagnosed, were still struggling to understand their situation. Practitioners described multiple layers of need among infected people and their families, compounded by traumatic encounters with healthcare providers and the wider system, and the impacts on mental health. They discussed the likely impacts of the Inquiry, which was ongoing at the time of data collection, highlighting that revisiting memories or discovering new information could cause renewed trauma.

[T]he people who survived buried their stories for many, many years. The trauma of a parent who in effect feels they've killed a child can sometimes only be dealt with by saying, ‘I'm not going to talk or think about it,’ and then the Inquiry has come along, and it's raised all these issues. And telling that story again and again is triggering and retraumatising.

[305]

There was an expectation among practitioners and some infected and affected people that there would be increased need for support after the Inquiry.

### 3.2 Barriers to Accessing Psychological Support

As noted, our interview analysis identified three themes, which we discuss in turn. We illustrate identified issues with selected quotes, with a wider selection presented in Table S1.

#### 3.2.1 Personal and Social Barriers

Personal and social barriers ranged from familial obligations, challenges in acknowledging the need for help, stigma, shame and secrecy, to perceptions of social roles.

##### 3.2.1.1 Family Responsibilities

Some participants reported prioritising familial responsibilities, such as bringing up children, over support‐seeking for their trauma. Some believed that opening up would stop them from being able to function.

##### 3.2.1.2 Difficulty Recognising or Acknowledging the Need for Help

Some people did not recognise their need for help and had felt alone and isolated, thinking they had to ‘get on with life’. Some participants with haemophilia described this attitude as a form of resilience, which may have deterred them from seeking support. Often people were not aware of support, and where they were, there was uncertainty whether it could help.

I tried to deal with everything on my own. A year ago, well last year, I was just like, ‘I really need—the Inquiry's not finishing. I'm not coping. It's affecting me still. I'm still getting flashbacks, I'm still getting the … you know, I need help with this. I'm having endless—more diagnoses linked to that. I'm being treated differently or badly by medical people who treated me before they'd found out the diagnosis and then wouldn't see me until after treatment, and their attitudes have changed to me.’ So I needed help.

[105]

##### 3.2.1.3 Stigma, Shame and Secrecy

Stigma, shame or not wanting to burden others were common reasons for infected individuals to not discuss their diagnosis or the infected blood scandal with anyone.

He was given his diagnosis basically and we were sent away. There was nothing, there was no support. And because it was such a stigmatised thing […] At that time, it was pretty horrific. We never told anyone, so we just lived in this little bubble of secrecy. And to be honest that's what's done the damage, […] is not being able to tell family, friends, for them to understand why you're behaving the way that you are and why the situation is what it is with you.

[102]

For many, the stigma of their illness made them afraid to ask for help for fear of more people finding out about their diagnosis. There were reports of discrimination. Stigma was mentioned as a particular challenge in minority ethnic communities. Several participants continue to feel anxious in healthcare settings due to ongoing stigma and repeated questioning about the origins of their infection. One participant described receiving death threats and abuse and eventually having to move house.

Even where participants had shared their worries with loved ones, discussions about infection and its impacts were often avoided or seen as unhelpful, creating additional burdens and reluctance to seek support. Practitioners cited recurring themes of stigma, secrecy, difficulty forming relationships and the consequent emotional impact and isolation.

A lot of them basically will live in complete secrecy—completely. Even with regards to partners. So, they don't actually really have support. It really limits some of their social interactions with others. And it's also important to say that a lot of them, it's coming from an older generation and part of being affected by the scandal, they don't even share their haemophilia status because it's known among the groups, the link between the haemophilia and HIV.

[312]

##### 3.2.1.4 Perceptions of Social Roles

About half of the men in the study felt that societal expectations of their role as providers prevented them from seeking support. They expressed reluctance to discuss their experiences with family or friends, viewing sharing information as risky. This could lead to individuals compartmentalising feelings about their infection, despite recognising that this behaviour may be unhelpful.

#### 3.2.2 Structural Barriers

Structural barriers to accessing psychological support included: lack of appropriate support, a fragmented referral system and long waiting times, professionals' lack of knowledge about the contaminated blood scandal, difficulty finding suitable practitioners, and limits on the number of therapeutic sessions. These barriers frequently overlapped, compounding the difficulties for individuals seeking support.

##### 3.2.2.1 Lack of Appropriate Support

Most participants reported never being offered psychological support and being left to ‘get on with stuff’. There were reports of distressing situations, such as following a suicide attempt or after bereavement, when no support was provided.

It was just me, fighting to get him a transplant. It was really hard. Yet the National Health Service had given him this [hepatitis C]. So, by the time they made their minds up he was too ill. […] It'd have been lovely to have spoken to somebody. [….] And [our daughters], of course, they didn't realise he was dying. It was awful.

[202]

Some people described trying to find a suitable therapist or access mental health support through their GP, who often seemed ignorant of their situation and needs. A few reported being offered ‘counselling’ in the form of a leaflet, generally about HIV, hepatitis C or bereavement when their loved one died. Some who had contacted mental health services or therapists felt misdiagnosed or treated without a proper understanding of their experiences.

[B]ecause of this and all the stress I was put under—well, I tried to commit suicide. I took overdose. […] And, yes, no, I didn't get counselling for that. […] No, they just said it was my mood. I was trying to get attention.

[121]

People shared experiences of being referred to inappropriate services, including a sexual health clinic. One resorted to attending a support group for drug users. Some participants had set up their own support groups or sought support outside the NHS, such as a hepatitis C group run in a local prison.

Practitioners highlighted the potential harm of inappropriate or generic psychological support, which could discourage people from engaging in further treatment. They stressed the need for tailored, long‐term and in‐depth treatment.

##### 3.2.2.2 Fragmented Referral System and Long Waiting Times

Referral pathways to mental health care were experienced as complicated and convoluted. The challenges of finding support were compounded by long waiting times. By the time people had navigated the referral process, support was often deemed too late or no longer suitable for their situation and needs.

To start with, I had to fight to get [mental health support]. You know, I had to sit at my doctor's and explain why I would need that, […]. Also, once I'd had it, I had to go through the process of (1) waiting ages, (2) having to go and see one service who then referred me to another service who referred me to a third service, to the point I'd got to, ‘OK, I've not got to see somebody who I think is going to listen to me, ask my full problems and history,’ and [the counsellor] actually turned around to me and said, ‘Oh my gosh, I'm getting stressed listening to it. I don't know how you cope’.

[105]

For some, the prolonged wait led to their condition deteriorating, resulting in relationship breakdowns and a sense that help would not be accessible. Some people with pre‐existing mental health conditions also did not receive appropriate support.

##### 3.2.2.3 Professionals' Lack of Knowledge About Contaminated Blood

Lack of knowledge about the infected blood scandal among professionals and the need to repeatedly explain their story were significant barriers to accessing care. Counsellors, therapists, GPs and medical specialists were frequently unaware of the contaminated blood scandal and were described as ‘ignorant’ and ‘damaging’.

I remember going to hospital appointments with [my husband]. One of them might have been a dermatology appointment, and the doctor would kind of look at his notes and go, ‘Oh, you've got hepatitis C. So, are you still an intravenous drug user?’ And I remember just practically levitating off the chair with anger. [husband] was always very calm and would just say, ‘No, it's through a contaminated blood transfusion,’ and then you'd get the response of, ‘Well, is that proven?’

[206]

Some experienced frustrating and detrimental cycles of beginning counselling, using up sessions, waiting for new referrals and having to repeat their story at the new cycle. One participant had to repeat their story to over 20 professionals without finding an appropriate practitioner. The need to educate professionals about the infected blood scandal was perceived as a waste of counselling time. Some found the effort of explaining so exhausting that they gave up seeking support.

##### 3.2.2.4 Difficulty Finding an Appropriate Practitioner

Closely linked to the above was a reported struggle to find a suitable therapist or counsellor more generally. People were often unsure of where to begin their search or whom to approach; some encountered professionals who deemed their cases too complex or traumatic and turned them down. Participants described therapists being overwhelmed by their stories, with one recalling a therapist breaking down in tears, unable to handle the conversation. Another participant described a counselling session they attended with their partner:

And at the end of an hour which I was paying for, [the counsellor] said ‘Well, that's all very, very complicated and I don't think I'm in a position to help you.’ And [they] wouldn't see me but I still had to pay for that consultation; £150 it cost me for somebody who said my problems were too complicated, too whatever for [them] to deal with.

[116]

Such experiences exacerbated feelings of frustration and anger with ‘the system’ that repeatedly failed them. Practitioners confirmed the difficulties people faced when trying to find appropriate therapists. They also noted it was difficult for individuals to know what kind of support might be useful to them.

Where to even find somebody? Again, nervousness of being able to go forward to a professional and not really knowing where to start. Because unless—it's a very daunting thing, what do you do, randomly search for a psychologist in your area, you don't know whether you need counselling, whether you need psychological support, whether you've got [post‐traumatic stress disorder]. You don't know whether it's anxiety, whether it's generalised anxiety, whether it is depression. You've got to almost quantify it yourself.

[303]

#### 3.2.3 Barriers Related to the England Infected Blood Support Scheme

Barriers related to the EIBSS ranged from insufficient information; the complexity of applying for funding for psychological support, including proving the need for support; restrictive criteria and inadequate funding, to a lack of support in finding a suitable practitioner. Participants found it difficult to repeatedly advocate for themselves, and some experienced feeling abused, especially when their applications for funding were rejected.

##### 3.2.3.1 Inadequate Information and Support

Participants who knew about the EIBSS funding for psychological support appreciated its availability. However, almost half of the study participants were unaware that such funding was available. Some learned about it through casual remarks from medical staff, chance encounters or social media. Others first heard about it at the Inquiry or during these research interviews, despite being registered with the EIBSS. Participants reported that the EIBSS had not actively encouraged the use of this financial help.

While a few people found the EIBSS payment helped them access psychological support, more participants were unsure about what types of therapy were most appropriate. The EIBSS was regarded as unhelpful, and participants commented on a lack of guidance on the website or from the team. One participant described it as ‘impossible’ to find a therapist, recounting that they had approached eight counsellors from the EIBSS list, all of whom refused to take them on. They found the process so stressful that they felt they ‘*wanted to throw [themselves] in front of a truck because everybody was saying no’* [105].

##### 3.2.3.2 Complexity of Applying for and Accessing Funding for Support

Very few people found the EIBSS process for applying for funding for psychological support easy, and many needed help completing it. Most found the system overly complicated and traumatising, and advocating for themselves difficult. Participants cited challenges with paperwork, navigating the EIBSS website, and repeated requests for justification, referrals and explanations.

And if you trail and trail around their complicated website, which is complicated for someone who's looking for the first time, who's not sure what anything means, we are offered £900 but then you've got to find the person and you don't want all that, you're tired, your body has had enough.

[120]

The need to prove eligibility for support and obtain medical records, often at personal expense, caused frustration. Several participants were unable to obtain their medical records required to make the case for funding, or they had difficulty providing evidence because their doctor or therapist refused to support their claim.

But the [hospital], you're asked to write to them. They took about 3 days to write back and say, ‘We don't have your records because we destroy all the records after 8 years.’ Well, hang on a sec: how are you supposed to get any kind of compensation, then, if your—in fact, your records have been destroyed by the very organisations that's asking for it? That's dreadful.

[104]

Participants also cited restrictive criteria for support, both in terms of eligibility and the nature of support provided. We heard reports of support being rejected for loved ones who had been profoundly affected by their situation.

I tried to get counselling for my mum and they rejected her; they said there was no funding. […] My mother has been there every step of the way; she's had to deal with haemophilia, AIDS, Hep C, CJD [Creutzfeld‐Jakob Disease]; she was there when I was told I was going to die a very horrible death and end up a vegetable on life support. And at the moment, my mum's not even recognised as somebody [who is affected].

[110]

Practitioners noted that the eligibility criteria for psychological support payments were not well understood, and some individuals might be unaware they are eligible. As a result, some participants felt that outcomes of the application process depended on chance and their ability to articulate their case. Where errors were made, the burden of proof was on the claimants to correct, leading to a further breakdown in trust, with some individuals deciding to forgo applying for funding altogether.

##### 3.2.3.3 Limits on the Available Funding for Psychological Support

Where people managed to access the £900 payment made available by EIBSS, they often found it inadequate and dehumanising. Several participants felt that the limited number of sessions covered by EIBSS was insufficient for meaningful treatment and not very helpful.

The limit on the number of sessions added stress, as people felt pressured to rush through their trauma processing.

They're always telling you how many sessions you've got left and I find that really difficult and almost offensive because I know when they say ‘right, that's it. It's over,’ well, it's not over [laughs]. […] You don't get open‐ended emotional support and to me, that's just a travesty and it's Orwellian in terms of the situation that I'm supposed to be getting support for. Everybody talks about ‘are you getting any support?’ And I say ‘yes, but for 6 weeks.’ [laughs] 6 weeks into 35 years of my diagnosis, I get some psychological support and then I don't.

[116]

Study participants reported how quickly their funds were exhausted, forcing them to stop counselling to avoid financial strain. Practitioners also noted that the limited number of sessions was inadequate for addressing the profound impacts of infection, especially for people who have been dealing with it for decades.

##### 3.2.3.4 Devaluing Experience of Applying to the EIBSS

The feedback on the help received from the EIBSS was overwhelmingly negative. Several participants experienced feeling abused, especially when their EIBSS applications were rejected after huge efforts. Many felt mistreated, and some reported being denied funding for approved interventions. Many expressed desperation about being unable to access care because of EIBSS, compounding their trauma.

I've never had any support from them [EIBSS]. In fact, I've had the opposite; they've treated me again like a criminal, I'm unworthy, you have to jump through all these different hoops, you know. […] Instead of them being them and us, seen as we are nothing more than trying to fleece the system, you know, benefit frauds. And that's how I have always felt, that I have to keep justifying my existence to these people who are apparently here to support me. And I know that is across the board, I mean, everybody hates EIBSS, they really do, because it's just another scheme that you've got to go cap in hand to and justify and, you know, I've had nothing out of the EIBSS, nothing at all.

[110]

Overall, these experiences contributed to general feelings of abuse and distrust in medical professionals, the NHS and public sector bodies generally, and the EIBSS specifically. Practitioners also noted that this distrust in institutions and services prevented people from accessing psychological support.

This is a persistent, for many, attachment‐based trauma, multiple hospital visits where there was fear and uncertainty, the sword of Damocles hanging over them about each treatment, ‘I need the treatment, but is it going to infect me?’ The anger and the mistrust of NHS England, doctors, who knows, but somewhere. And then all the ‘I can't tell anyone this because they'll think things about me and look what the news is saying about.…’

[314]

## 4 Discussion

This study found a lack of effective psychological support available for individuals infected and affected by the profound consequences of HIV and/or hepatitis C infections from contaminated blood and blood products in England. The results highlight the multiple and significant barriers that individuals encountered when seeking support and emphasise the urgent need for an effective and accessible support system.

### 4.1 Summary of Key Findings

Infected and affected people have faced unprecedented challenges, navigating the consequences of their infections for a long time. This prolonged ordeal has had profound impacts on the mental health of infected individuals and their families. Most have not received psychological support, and half were unaware of the EIBSS funding. The few who accessed support found it inadequate or ineffective, with some giving up on finding help. Barriers they encountered included personal and social issues, such as family obligations, isolation and reluctance to share their diagnosis due to shame, stigma and discrimination. Numerous structural barriers compounded these challenges, including difficulty finding a suitable practitioner, limitations on the number of therapeutic sessions, and professionals' lack of knowledge about the contaminated blood scandal. Participants faced further challenges when accessing the EIBSS funding for psychological support. Many cited a lack of information and a complex application process. They struggled to prove eligibility and experienced feelings of abuse. Restrictive criteria and insufficient financial support were deemed inadequate for meaningful treatment.

Practitioners highlighted the inadequacy of the current support system in addressing the profound impacts of infections, as well as the emotional impact and isolation many individuals experience. They confirmed the substantial need for psychological support within the infected and affected communities, anticipating an increase after the Inquiry.

### 4.2 Comparison With Existing Literature

Our findings are consistent with the accounts of infected and affected individuals who shared their experiences with the Inquiry [7, 22, 23]. Poor quality of life and high rates of depression among people with hepatitis C and/or HIV are well documented, with co‐infected individuals experiencing worse impacts [24, 25]. Our study adds to the existing evidence by illustrating the ongoing, significant need for psychological support within the infected and affected communities in England, as well as the challenges in accessing adequate services. These needs were expected to increase after the Inquiry's conclusion in May 2024. The Haemophilia Society warned at the outset of the Inquiry that the lack of appropriate support was putting more lives at risk [26].

Research on the psychological support needs of individuals impacted by the contaminated blood scandal is limited. Although legal cases related to contaminated blood in other countries have been closed [27], significant challenges remain, as people continue to live with the consequences of their infections. This study highlights the critical need for systems that provide support, recognise trauma, and address mental health challenges following major incidents or crises.

### 4.3 Strengths and Limitations

We successfully recruited a diverse range of participants across age groups and regions in England, with varied experiences of psychological support, including those funded by the EIBSS or other sources, and those who received no support. The higher proportion of women in our sample compared to the UK population likely reflects the challenges in recruiting men to health studies [28]. The predominance of White British participants probably reflects the demographic composition of the infected and affected communities. However, it is unclear whether the sample accurately represents the wider group of EIBSS beneficiaries, as comprehensive data on the total infected and affected population are lacking.

Also, several methodological limitations should be considered. Our recruitment strategy may have introduced a selection bias, despite purposive and snowball sampling efforts to ensure diverse representation. To strengthen methodological rigour and trustworthiness, we held regular reflexive team discussions on emerging themes from interviews, our responses and our developing analysis, to ensure balanced perspectives between infected individuals, affected family members, and mental health practitioners.

Furthermore, as the interviews were conducted while the Inquiry was ongoing, the EIBSS has since amended some processes, which may not be reflected in the participants' responses. Finally, time constraints restricted the number of interviews conducted, potentially narrowing the study's scope. Despite these limitations, the consistency of themes across participant groups and their alignment with evidence from the Inquiry support the credibility of our findings.

### 4.4 Implications for Policy and Practice

To address barriers to supporting people impacted by contaminated blood, relevant agencies must consider the current support needs of these communities by drawing on the expertise of established mental health experts, support organisations, and service models across the United Kingdom. Involving impacted individuals in designing and implementing services will be crucial in rebuilding trust and confidence in the system, particularly regarding the EIBSS.

Establishing an effective support system requires a comprehensive approach without complicated application processes. It should integrate proactive measures and incorporate navigators to facilitate access to diverse services. Services should be accessible to a wider range of individuals, offering multiple access routes, including self‐referral options, and using flexible service delivery models to facilitate easy re‐entry and continuity of care. Specialised settings should provide comprehensive assessments and a range of therapeutic modalities to meet the diverse needs of impacted individuals. Mental health practitioners should be not only qualified but also experienced and sensitive to the unique challenges of this community. Implementing quality assurance mechanisms would ensure that services meet established standards and provide effective support.

## 5 Conclusion

The contaminated blood scandal is one of the most tragic episodes in UK public health, yet its consequences have not been addressed. Many infected individuals and their families have suffered profound physical, psychological, social and economic impacts, most without adequate psychological support. They have endured multiple layers of trauma and injustice, often for decades: from initial infection, mistreatment by healthcare services and suffering in isolation, to facing numerous barriers when trying to access support. Current psychological support systems in England, both public and private, fail to meet the needs of infected and affected communities. Our findings show a substantial and increasing need for accessible, effective and personalised services. This desperate need must be addressed urgently by providing a long‐term, specialist support service, reaching out to all impacted individuals, without burdensome requirements. This will represent a significant step for the government to begin addressing the harm it caused.

## Author Contributions

**Eva Cyhlarova:** conceptualisation, investigation, methodology, formal analysis, writing – original draft, writing – review and editing. **Jessica Carlisle:** methodology, investigation, writing – review and editing, formal analysis. **Emily Warren:** formal analysis, writing – review and editing. **Martin Knapp:** conceptualisation, methodology, writing – review and editing. **Ellen Nolte:** conceptualisation, methodology, formal analysis, writing – review and editing, supervision, investigation.

## Ethics Statement

Ethical approval for this study was granted by the Observational/Interventions Research Ethics Committee at the London School of Hygiene & Tropical Medicine (LSHTM Ethics Ref: 28215).

## Conflicts of Interest

The authors declare no conflicts of interest.

## Supporting information

Cyhlarova Supporting File 1 Interview guides 200525.

Cyhlarova Supporting File 2 COREQ 200525.

Cyhlarova Supporting Table 1 200525.

## Data Availability

The data that support the findings of this study are available on request from the corresponding author. The data are not publicly available due to privacy or ethical restrictions.
