# Supplementary material for: ‘They Just Said It Was My Mood. I Was Trying to Get Attention’: Exploring Barriers to Psychological Support for People Impacted by Contaminated Blood in England
Source: Health Expect. 2025 Jun 10;28(3):e70317. doi: 10.1111/hex.70317 (PMC12149982; doi:10.1111/hex.70317)
Supplement: Supplementary file 2 — Cyhlarova Supporting File 2 COREQ 200525. [file HEX-28-e70317-s002.PDF]

Supplementary File 2: Consolidated criteria for reporting qualitative research (COREQ) checklist

Supplement to: 'They just said it was my mood. I was trying to get attention': Exploring barriers to psychological support for people impacted by contaminated blood in England

**Criteria**	**Detailed information**	**Where is the information stated?**
**Domain 1: research team and reflexivity**		
Personal characteristics		
1. Interviewer	EC, JC and EN conducted the interviews	4
2. Credentials	All authors hold a PhD degree and have extensive experience of conducting qualitative research	n/a
3. Occupation	EC: Senior Research Fellow<br>JC: Research Officer<br>EN: Professor of Health Services and Systems Research<br>EW: Assistant Professor<br>MK: Professor Health and Social Care Policy	n/a
4. Gender	The research team is comprised on 4 females and 1 male researcher	n/a
5. Experience and training	All researchers have extensive experience of conducting qualitative health research. EC and JC have significant expertise in conducting interviews on complex and sensitive subjects<br>EN has significant experience of conducting elite interviews with healthcare professionals and policymakers	n/a
Relationship with participants		
6. Relationship established	Members of the research team had no relationships with participants prior to the study. It was important that the interviewers were seen as unbiased enquirers rather than having a particular opinion about the topic.	n/a
7. Participant knowledge of the interviewer	The interviewers introduced themselves to participants stating that they were not medical professionals, etc., and described the purpose of the study and answered any questions about the project and those involved in it.	n/a
8. Interviewer characteristics	The interviewers were not clinicians, but were familiar with current support available, had read available literature and other materials on the contaminated blood scandal, and consulted with organisation providing support.	n/a
**Domain 2: study design**		
Theoretical framework		
9. Methodological orientation and theory	The analysis used mainly a thematic approach, incorporating elements of grounded theory, such as constant comparison and the examination of deviant cases.	
Participant selection		
10. Sampling	Participant recruitment used snowball and purposive sampling.	4

11. Method of approach	We used two approaches to recruit a sample of infected and affected participants:<br>1. A total of 36 expressed interest in the study via the support organisations and 24 were interviewed.<br>2. A total of 366 EIBSS survey respondents indicated their willingness to participate in further research. We contacted a purposive sample, seeking to capture people from a range of socio-demographic backgrounds with a range of experiences in terms of EIBSS payment and psychological support, as shown in Table 1. Of 63 invited, 28 agreed to be interviewed.<br>Potential mental health practitioners and experts were identified from relevant organisations' websites, the authors' professional networks, as well as recommendations from interviewed participants.	4
12. Sample size	Infected and affected participants: 52<br>Mental health practitioners and experts: 14	4
13. Non-participation	We did not interview two participants as they were not from England and therefore not eligible to receive EIBSS support. Ten people who initially expressed interest decided not take part.<br>We were unable to arrange interviews with two mental health experts due to time constraints.	4
Setting		
14. Setting of data collection	Interviews with infected and affected participants were conducted online (n=31), in-person (n=12), or telephone (n=9) according to participant preference. Nine face-to-face interviews were conducted a private meeting room on the university campus, and 4 participants were interviewed in their homes.<br>Mental health practitioners and experts were interviewed using MS Teams.	4
15. Presence of non-participants	None	
16. Description of sample	Presented in the results section and in Table 2.	5
Data collection		
17. Interview guide	Interviews followed a semi-structured topic guide based on consultations with experts and support organisations. For infected and affected individuals, we explored the impact of their infection(s), their views and experiences of psychological and other support, and their experiences with the England Infected Blood Support Scheme (EIBSS). We also collected demographic information.<br>Interviews with mental health practitioners and experts explored the current need for psychological support, barriers to access, and the characteristics of effective support services. The topic guide was adapted to reflect differences in services across the UK.	Supplementary file
18. Repeat interviews	We did not perform repeat interviews.	n/a
19. Audio/visual recording	All interviews were recorded and later transcribed.	4
20. Field notes	The interviewer wrote brief notes after each interview.	

21. Duration	Interview times varied but lasted an average of 60 minutes (for infected and affected participants) and 52 minutes (for mental health practitioners and experts).	4
22. Data saturation	Due to the complex and varied nature of the issues and participant experiences, data saturation was not applicable. However, we are confident that we identified the key issues for both samples within the available timeframe.	n/a
23. Transcripts returned	Transcripts of interviews were offered to all participants for review and provided to 5 participants who expressed interest.	n/a
**Domain 3: analysis and findings**		
Data analysis		
24. Number of data coders	Data were organised and managed in NVivo 2020 and coded by EW with assistance from the study team.	4
25. Description of the coding tree	The initial coding framework was informed by the research questions and objectives. This framework was then expanded, refined, and restructured into a parent-child node hierarchy after an initial phase of inductive coding, followed by axial coding to explore relationships between in-vivo codes. The analysis used a thematic approach, incorporating elements of grounded theory, such as constant comparison and the examination of deviant cases.	
26. Derivation of themes	The team met regularly to discuss and identify patterns of shared meanings or 'central organising concepts' using an open and iterative approach (Braun, Clarke & Rance, 2014; Braun and Clarke, 2019). JC and EC led discussions about the analysis of interviews they had conducted with infected and affected people and EN led on discussions about the analysis of her interviews with professionals. The process of developing, synthesising and contrasting themes continued until the final analysis (Braun and Clarke, 2018), leading to a rich interpretation of the meaning and experience of the contaminated blood scandal (Byrne, 2021).	4
27. Software	NVivo 2020 was used for coding.	4
28. Participant checking	n/a	n/a
Reporting		
29. Quotations presented	Quotations are presented and identified in a manner protecting participant confidentiality.	5-11
30. Data and findings consistent	There is consistency between the data and the findings reported.	5-11
31. Clarity of major themes	We described in the text the main barriers to assessing support. The themes were consistent across the two samples (infected/affected people and practitioners/experts).	5-11
32. Clarity of minor themes	See 31	5-11
