# Supplementary material for: ‘They Just Said It Was My Mood. I Was Trying to Get Attention’: Exploring Barriers to Psychological Support for People Impacted by Contaminated Blood in England
Source: Health Expect. 2025 Jun 10;28(3):e70317. doi: 10.1111/hex.70317 (PMC12149982; doi:10.1111/hex.70317)
Supplement: Supplementary file 3 — Cyhlarova Supporting Table 1 200525. [file HEX-28-e70317-s003.pdf]

Supplementary Table 1. Barriers to accessing psychological support: selection of quotes from infected and affected individuals, and practitioners and experts

Supplement to: ‘They just said it was my mood. I was trying to get attention’: Exploring barriers to psychological support for people impacted by contaminated blood in England

	Infected and affected participants	Mental health practitioners and experts
**Personal and social barriers**		
Family responsibilities	*And I still didn’t know anything about Hep C, didn’t have any friends, but there was a local meeting going on and I went to it, and I like everybody seemed to know each other, everybody knew what was going on, they were active, and I just collapsed in a heap. I think I had a breakdown then. And I again applied to the money, and I said that the – because I gave my interview then, and I’d never thought about how I was feeling or whatever, I just got on with it, had to, I had three young kids.* [107]	
Difficulty recognising or acknowledging the need for help	*So, then she [counsellor] said, well, would you like to talk about that? And of course, I didn’t know what to say because nobody’s ever asked me before, nobody’s ever said, well, you’ve got this, what was the impact on your life? And I’d never thought about it because I’d been – I just got on with my life because I come to the conclusion very, earlier on, I was going to die.* [107]	
Stigma, shame and secrecy	*Because we’d had anonymous death threats and all the rest of it and abuse and we’d had to move house, I didn’t want to be round anybody. I didn’t want to have to sit around other people. I was almost scared to speak in the rooms in case somebody might overhear.* [105]<br><br>*I didn’t think there was support our there. Because we’ve kind of been treated like lepers and that’s what it felt like, I didn’t like to put my hand up and say – because I’m afraid what people will say.* [139]	*This is years of secrets within families. Shame that comes alongside secrets that have been held for a long time and fear about being exposed in a community or in a family, and how that manifests in someone. The trust that we talked about on healthcare level – it’s an interpersonal challenge. So people have often spent years without relationships or distant or isolated. And obviously, there’s an emotional impact of that over time.* [311]

Perceptions of social roles	*And I realised at that point that I'd built these walls and didn't let anybody in, and psychologically. I know I'm a bloke and we don't talk about stuff and you know, it is I think slightly different for men because we just kind of get on with stuff. But it's not the best way as I have found out. And I spoke to a couple of guys and they'd done exactly the same thing.* [108]<br><br>*And that was in a time when A) you didn't talk about mental health and B) you definitely didn't talk about mental health as a man, especially in a macho working environment like that.* [123]	
**Structural barriers**		
Lack of appropriate support	*My sons have tried to kill themselves over this. They were under [Child and Adolescent Mental Health Services, CAMHS] at the time, but CAMHS did not understand it. They couldn't grasp it and, of course, they outgrew CAMHS and were transferred to the adult service. The adult services didn't take notice of what was actually being said and they were just like, "Come on, why don't you go to a youth club?" and it's like, "Boom, you haven't listened". I think that's the biggest thing I can say. People need to listen to what we're saying, not think they know what we're going to say to their question.* [105]<br><br>*I phoned the local Talking Therapies for the NHS and was told that obviously I can't do anything through the NHS, I would have to go to a private therapist, and it was them that sent me the details of all the local people. But there's so many on there, I just wouldn't know who to pick or which way to go with it. So, to be honest, that [EIBSS] form is still sitting here, so I, and also my family have not pursued it in any way because it just seems such a barrier to go through.* [213]	*And for me, it is a crying shame that people might think – that [generic support] might be their experience of therapy, because clearly they're needing something that is much more tailored, long term, in depth and very knowledgeable about their specific pain.* [308]
Fragmented referral system and long waiting times	*My wife's saying, "Maybe you should move out?" You know, that's how bad things had got, and she wasn't wrong; these were the options we were facing. And there was no point going back to the GP because I was on a waiting list that seemed to be endless, psychological support from the GP. And there was no point going – I'd been to A&E with, you*	

	know, a breakdown, taken in by ambulance and that didn't really get far. [116]<br><br>*They locked me up in [psychiatric care] because the OCD was so bad, but the OCD was saying, "Well, if you don't do this, your hepatitis will come back. If you don't do this, someone in your family will die."* [134]	
Professionals' lack of knowledge about infected blood	*He [my son] tried to get that for me, and this form to sign, it had to be signed by your GP or by the consultant, and it was amazing that the GP never heard of that form. He didn't know anything about it, and we were going to and fro, and we were driving there, and they wouldn't sign it. That needs to be addressed. […] The specialist wouldn't sign it; they said the GP has to sign it.* [142]<br><br>*I think I've had one or two support sessions over the years, and I've not found any of them to be helpful in any meaningful way. So, I've spent all my time explaining to the person what the inquiry is all about and I felt that they probably got – well, not even probably. They got more out of the session than I did.* [123]	
Difficulty finding an appropriate practitioner	*Only in later years, say in the last four/five years, I've tried other people. Another woman who just sat there looking so absolutely appalled and out of her depth. I was worried about her, which kind of is like a theme really. Well-meaning, nice, middle-class ladies that don't believe in state sanctioned cover-ups and it's too much for them to cope with […] So I don't blame them, but it's been an utter waste of my time and money and just made me feel worse.* [111]	
Limits on the number of sessions	*I don't know if they relayed that back to my psychiatrist and if you were sent to a psychologist, he expected you to be right in six weeks. Then it's goodbye. In COVID and for some reason even my GP can't understand why my psychologist discharged me*. [141]	*It's very hard to get rid of behaviour that you've been living with for six months never mind six years, or it could be 40 years, 50 years for some people. So, they're really entrenched, and I don't think a few counselling sessions is really going to help with that. I don't even know if two years of psychotherapy is going to help with that either, but it feels to me that it should be more weighty.* [302]

Barriers related to the England Infected Blood Support Scheme		
Lack of information about funding and support	*I really wish, back then, I would have known about the Hepatitis C Trust and that kind of thing, because then I could have spoken to them or – you know, and I know like the EIBSS thing now you can get counselling. I've only found out about that in the last two months. You know, like you can get counselling. So, a lot of my life I've just been dealing with this the best I can. Yes, it's not been easy.* [203]	
EIBSS' complicated application process	*Yes, it's like getting a quote from them that they can then agree, the same as you have to apply for anything else, you have to get two quotes. It's doing that as though it's like a washing machine. It's horrible.* [105]<br><br>*No, I've never applied for anything from EIBSS, ever, and the reason why is that I can't be – excuse my French now – arsed to go around all their pathetic forms and getting quotes for this, quotes for that. Like I said to him, "We're not going to get any better, we're not suddenly, going to wake up one day and go, "My God, my liver's perfect now," and I can go back to work."* [120]	
Need to make the case for support	*But now my therapist has turned around and said, "I'm not going to do the thing you need me to do in order for you to finally get the thing you've fought so hard to get justice for." [212]*<br><br>*It's my understanding that the psychoanalytic psychotherapy, which is one of the therapies that is on the EIBSS list – it's that kind of therapy who – their thing is that you should pay for your own treatment.* [212]<br><br>*I kept applying because I thought this is ridiculous. And I was refused three times. So, I gave up, I thought I can't fight it.*[107]	*I think the criteria for accessing support perhaps excludes some of the people who have been affected. And there's the ongoing issue which is coming up […] quite a lot, that people who didn't even know or who have found it hard to get proof. You know, the whole issue of testing and getting hold of medical records, and I think for some people the barriers are just so great that they just think it's just not worth it.* [305]
Limitation of EIBSS' criteria for support	*So I wrote asking, "You do this allowance for counselling. Could I please apply it to this? Because this is working for me," and was told no. It's not counselling.* [109]<br><br>*[A friend] was having really successful online sessions with an Australian woman who she highly recommended. So I thought, "Oh*	*[A]nd then there's the people where actually they have no access at all to anything through EIBSS because they have no connection. They're a sibling, they're a carer, they're a child of. They're not connected to the support scheme. They don't get the notifications. If they're not*

	well, I'll see if I can get funding for that because she is a counsellor." But because she wasn't registered with the British Counselling Association, that was also impossible. So it's been one of those things where I've had to go my own way. [109]	*members of [organisation] they don't even know that they can access that, and that's been quite recent as well.* [303]
Inadequate funding	*I just think it works up to a point or it does something up to a point. But then I'm wasting the money, because all I'm doing is starting something off, and it can't be finished. And I can't support it financially on an ongoing basis unless it is subsidised in some way.* [210]	*And the discretionary payment doesn't amount to very many sessions. I think it equates to something like maybe twelve? So we're still in the realm of short-term work. […] It can seem tokenistic, because what's twelve sessions, really? I think there needs to be something ongoing.* [308]
Lack of support from EIBSS to find suitable treatment/practitioner	*So they would fund it, but I understand it's just impossible to find someone who actually can do the job.* [105]	
Devaluing experience of applying to EIBSS	*As a group, we all hate them [EIBSS]. [...] And we don't like the process, we don't like the outcomes. Clearly, we're biased but we think we've got a good reason [laughs] to be biased. If you're offered support and then find you don't qualify, it's really difficult. But it's just window dressing and it sounds brilliant until you try and get it.* [116]<br><br>*If I phone up right now, I won't get an answer to anything because you speak to them, they have to email and find out stuff and then they'll come back and give you some old wishy-washy answer. So you would never really get anything or it's all done by email. There's people there who I do think some of the people there do, sort of, care but because they're holed inside, they can't do anything so it isn't support, it isn't care, there's no compassion, there's no understanding. And we're talking about really vulnerable people who through no fault of their own, something happened, phone up and say "I need some help now," and nothing will happen.* [110]	*There's a huge, huge amounts of loss within that community. And they are quite bitter, actually, quite difficult to reach sometimes because they don't trust that we're going to support them, we have to earn their trust back.* [307]
